# Blow Pipe

**Published:** 1853-07

**Authors:** J. B. Williams

**Affiliations:** Dentist; Monongahela City, Pa.


*Blow Pipe.* By J. B. Williams, Dentist, of Monongahela City, Pa.

A permanent Blow Pipe, which produces a constant, steady blast, of any desired strength, always ready for use, costs but little and nothing to keep it up.

It consists of three sheet zinc cylinders, placed one within another, thirty-six inches in length. The largest cylinder is twelve inches in diameter; the inner, smallest-cylinder is 11½ inches in diameter; these are soldered together at the lower end, leaving a narrow space of about ¼ or ½ inch between them. This space is partly filled with water, and the third or middle cylinder, being closed at the upper end, is made to pass down into it; and neatly adjusted to the inner surface of outer cylinder, so as to pass between it and the inner one without much friction.

The smallest inner cylinder, is closed also at the upper end, except a small opening at the center, in which an ½ inch lead pipe is soldered, that passes out at the open end below; and is taken up through the end of the work bench; where a brass stop-cock, to regulate the blast, is screwed into the end of the pipe.

A short gum-elastic tube, 18 inches in length, is fastened to this, with the jet-pipe at its outer end; and is kept in any desired position by wires.

A strong cord passed over a six-inch pully, fixed to the ceiling, is attached to a head of wood 1¼ inch thick, placed within the upper end of the moveable cylinder, the other two cylinders being stationary, raised about twelve inches from the floor, upon this head, weights are placed. About ¼ inch of the outer edge of the moveable middle cylinder, is bent in upon the head of the wood to hold it firmly.

A valve—three inches in diameter, ¼ inch thick, its stem ⅝ inches in diameter, 3 inches in length, together with its seat are all of zinc, which I cast and had neatly turned in a lathe—is passed through the head of wood, and soldered to that of zinc within, having the valve inwards.

Around its stem, is a coil of wire, attached to its upper end, of sufficient strength to hold it up gently. Thus arranged, the whole is easily regulated with one hand, and then be at liberty.

I use a "*burning fluid lamp*." From its side near the top, is a flat tube with a close fitting slide and cap. It is two inches long, ¾ inch broad, ¼ inch thick, beveled at the end, the sides being perpendicular.

There is a small spigot at the bottom of the stationary cylinders and pipe, to let the water out in very hard freezing weather.

It may be necessary to state, that from the great power of the blast, (or pressure) thus produced, I had to place a cylinder of wood within the inner cylinder, to keep it from collapsing.

This, I suppose, cost about ten dollars, but, as I made a good deal of it myself, I cannot tell precisely.*

*Last summer, when on a visit up the river, we had the pleasure of seeing the above described, Blow pipe of Dr. William's. It struck us then, as a very convenient apparatus, and as we had never seen one of the kind, we requested a description for the Register. The jet is continued and plenty strong for any use in a dental laboratory.—[Ed. Reg.

